# Supplementary material for: Amino Acid and Essential Fatty Acid in Evacuation Shelter Food in the Noto Peninsula Earthquake: Comparison with the 2024 Simultaneous National Survey in Japan
Source: Nutrients. 2024 Dec 3;16(23):4185. doi: 10.3390/nu16234185 (PMC11643995; doi:10.3390/nu16234185)
Supplement: Supplementary file 1 [file nutrients-16-04185-s001.zip › nutrients-3310180-supplementary.pdf]

Supplementary File S1

Table The amount of non-essential amino acids in the 11 foods provided to the evacuation shelter in NPE.

Shelter 11 foods	Arg	Ala	Asp	Glu	Gly	Pro	Ser	Hyd-Pro
	mg	mg	mg	mg	mg	mg	mg	mg
A	508	449	739	3408	446	871	508	26
B	634	519	921	2422	556	742	493	37
C	1315	992	1777	3307	971	877	859	134
D	1419	1170	1965	3463	1004	904	973	100
E	1059	846	1588	2721	718	645	709	39
F	1072	892	1450	2763	783	699	732	95
G	1374	1213	2056	3483	1018	819	907	78
H	888	698	1168	2045	666	590	593	86
I	1188	998	1668	2745	916	770	788	110
J	928	711	1116	1998	707	588	598	124
K	825	630	1115	2065	615	529	530	86

abbrebiations, Arg: arginine, Ala: alanine, Asp: aspartic acid, Glu: glutamic acid, Gly: glycine, Hyd-Pro: hydroxyproline , Pro: Proline, Ser: serine. A, B, C, D, E, F, G, H, I, K, J: all alphabetical letters represent 11 different meals provided in the shelter where TMAT has medical activities in NPE.

Supplementary file S2

An application of “Duplicated combination” model to Set possible three foods set using Programming language

To explain the An application of "duplicated combination" model to set possible three food set using programming language in four application explanations. First, we explain the mathematical model of "duplication combination". Second, we explain the programming language. Third, we explain the generalization of the extensional application of this novel model to count combination numbers of three foods from any number of foods in any disaster victims. Finally, the fourth table calculated from the duplicated combination model gives an example of how much leucine each duplicated combination contains from all 11 foods.

# 1, Application 1- “Duplication combination” model

This mathematical idea of "duplicated combination" may refer to a special type of counting involving combinations where elements are allowed to repeat.

Combinations with Repetition

Combinations with repetition are often used to count the ways to select items from a set where duplicates of the same item are allowed.

Formula for Combinations with Repetition

If we have “n” different items and you want to choose “k” of them, with the possibility of repeating items, the number of possible combinations (also called multisets) is given by:

$$\binom{n+k-1}{k} = \frac{(n+k-1)!}{k! \cdot (n-1)!}$$

where:

- n is the total number of unique items.
- k is the number of selections to make.
- $\binom{\cdot}{\cdot}$ denotes the binomial coefficient.

In our present study, we had 11 types of food for the excavation shelter in Noto Peninsula. We choose any three out of 11, to calculate all the number of combinations 3 out of 11 using the above-mentioned "duplicated combination" model, the following mathematical equation is applied:

$$\binom{11+3-1}{3} = \binom{13}{3} = \frac{13!}{3! \cdot 10!}$$

Calculating this:

1. Calculate 13!13!13! only for the top three terms because 10!10!10! cancels out most of it:

   13×12×11=171613 ¥times 12 ¥times 11 = 171613×12×11=1716

2. Calculate 3!3!3!:

   3!=3×2×1=63! = 3 ¥times 2 ¥times 1 = 63!=3×2×1=6

3. Divide:

   $$\frac{1716}{6} = 286$$

So we could get the answer that there are 286 ways to choose 3 fruits from 11 types,

with repetition allowed.

# 2, Application 2- The programming language of the “Duplication combination” model to count possible three foods set from 11 foods in Disaster area

To calculate all possible combinations of three foods from 11 foods, when we use any statistical software such as Miscrosoft Excel software, we could use the following programming language written in blue ink:

Combinations = (n+r-1 / r) = (n+r-1)! / r! (n-1)!

Here, n=11 and r=3. So

Combinations = 13X12X11 / 3X2X1 = 286

The following commands were created in a statistical software such as Microsoft Excel:

a. Open the VBA Editor by pressing Alt + F11 .

b. Insert a new module by selecting Insert > Module .

c. Paste this VBA code into the module:

d. Run the Macro by going back to Excel and pressing Alt + F8, then selecting the GenerateCombinationsWithRepetition macro. This will populate the sheet with all the combinations in columns A, B, and C.

# 3, Application 3 - Generalization to extend to calculate all nutrient provision in large numbers of meals in evacuation shelters

For each combination obtained, the content of each amino acid and fatty acid can be

extracted from the database and inserted into each combination to calculate the amount of amino acid and fatty acid provided by each meal combination. In other words, this 'double combination' model can be extended to calculate the amount of other nutrients provided.

#4, “Duplicated Combination” model and its objective example for leucine

The mathematical "duplicated combination" model was used to calculate all the combinations of 3 freely chosen meals from 11 types of stored meals, allowing for overlap in their selection. This method was carried out using the spreadsheet software “Microsoft Excel” (Microsoft, USA) with the following programming language:

Determine the Formula: The formula to calculate the number of combinations with repetition is given by:

Combinations = (n+r-1 / r) = (n+r-1)! / r! (n-1)!

Here, n=11 and r=3. So

Combinations = 13X12X11 / 3X2X1 = 286

The Excel commands are:

a. Open the VBA Editor by pressing Alt + F11 .

b. Insert a new module by selecting Insert > Module .

c. Paste this VBA code into the module:

```
Sub GenerateCombinationsWithRepetition() Dim n As Integer, r As Integer Dim i As Integer, j As Integer, k As Integer Dim row As Integer Dim types As Variant n = 11 ' Number of types to choose from r = 3 ' Number of selections types = Array("Type1", "Type2", "Type3", "Type4", "Type5", "Type6", "T row = 1 For i = 0 To n - 1 For j = i To n - 1 For k = j To n - 1 Cells(row, 1).Value = types(i) Cells(row, 2).Value = types(j) Cells(row, 3).Value = types(k) row = row + 1 Next k Next j Next i End Sub.
```

Then, d. Run the Macro by going back to Excel and pressing Alt + F8 , then selecting the GenerateCombinationsWithRepetition macro. This will populate the sheet with all the combinations in columns A, B, and C.

In case of Noto Peninsula Earthquake (NPE) evacuation shelters

In the Noto Peninsula Earthquake (NPE) evacuation shelters, three out of 11 meal types were provided, but even in larger evacuation shelters where a large number of different meal types were lost prematurely, all combinations of three meal types can be calculated by entering the total number n mentioned above and the number of meals per day, r=3.

Generalization to extend to calculate all nutrient provision in large numbers of meals in evacuation shelters

For each combination obtained, the content of each amino acid and fatty acid can be extracted from the database and inserted into each combination to calculate the amount of amino acid and fatty acid provided by each meal combination. In other words, this 'double combination' method can be extended to calculate the amount of other nutrients provided.

4, An example of how to calculate the daily leucine intake

To calculate the daily leucine intake for the 11 meals listed from A to K and all 286 combinations are shown. The right end of each mela represents the total daily leucine intake. One mela represents the daily leucine intake for three meals for 286 meal combinations. This example could be extended to all nutrients for the large number of meals. The following table gives an example of how much leucine each duplicated combination contains from all 11 foods:

	Leucine
Meal	mg
A	785
B	790
C	1380
D	1603
E	1167
F	1224
G	1632
H	938
I	1338
J	918
K	864

Combination No.	Leucin Amount1	Leucin Amount2	Leucin Amount3	Daily Leucine intake
1	785	785	785	2354.1
2	785	785	790	2359.4
3	785	785	1380	2949.3
4	785	785	1603	3172.05
5	785	785	1167	2736.05
6	785	785	1224	2793.75
7	785	785	1632	3201.55
8	785	785	938	2507.19
9	785	785	1338	2907.19
10	785	785	918	2487.3
11	785	785	864	2433.01
12	785	790	790	2364.7
13	785	790	1380	2954.6
14	785	790	1603	3177.35

15	785	790	1167	2741.35
16	785	790	1224	2799.05
17	785	790	1632	3206.85
18	785	790	938	2512.49
19	785	790	1338	2912.49
20	785	790	918	2492.6
21	785	790	864	2438.31
22	785	1380	1380	3544.5
23	785	1380	1603	3767.25
24	785	1380	1167	3331.25
25	785	1380	1224	3388.95
26	785	1380	1632	3796.75
27	785	1380	938	3102.39
28	785	1380	1338	3502.39
29	785	1380	918	3082.5
30	785	1380	864	3028.21
31	785	1603	1603	3990

32	785	1603	1167	3554
33	785	1603	1224	3611.7
34	785	1603	1632	4019.5
35	785	1603	938	3325.14
36	785	1603	1338	3725.14
37	785	1603	918	3305.25
38	785	1603	864	3250.96
39	785	1167	1167	3118
40	785	1167	1224	3175.7
41	785	1167	1632	3583.5
42	785	1167	938	2889.14
43	785	1167	1338	3289.14
44	785	1167	918	2869.25
45	785	1167	864	2814.96
46	785	1224	1224	3233.4
47	785	1224	1632	3641.2
48	785	1224	938	2946.84

49	785	1224	1338	3346.84
50	785	1224	918	2926.95
51	785	1224	864	2872.66
52	785	1632	1632	4049
53	785	1632	938	3354.64
54	785	1632	1338	3754.64
55	785	1632	918	3334.75
56	785	1632	864	3280.46
57	785	938	938	2660.28
58	785	938	1338	3060.28
59	785	938	918	2640.39
60	785	938	864	2586.1
61	785	1338	1338	3460.28
62	785	1338	918	3040.39
63	785	1338	864	2986.1
64	785	918	918	2620.5
65	785	918	864	2566.21

66	785	864	864	2511.92
67	790	790	790	2370
68	790	790	1380	2959.9
69	790	790	1603	3182.65
70	790	790	1167	2746.65
71	790	790	1224	2804.35
72	790	790	1632	3212.15
73	790	790	938	2517.79
74	790	790	1338	2917.79
75	790	790	918	2497.9
76	790	790	864	2443.61
77	790	1380	1380	3549.8
78	790	1380	1603	3772.55
79	790	1380	1167	3336.55
80	790	1380	1224	3394.25
81	790	1380	1632	3802.05
82	790	1380	938	3107.69

83	790	1380	1338	3507.69
84	790	1380	918	3087.8
85	790	1380	864	3033.51
86	790	1603	1603	3995.3
87	790	1603	1167	3559.3
88	790	1603	1224	3617
89	790	1603	1632	4024.8
90	790	1603	938	3330.44
91	790	1603	1338	3730.44
92	790	1603	918	3310.55
93	790	1603	864	3256.26
94	790	1167	1167	3123.3
95	790	1167	1224	3181
96	790	1167	1632	3588.8
97	790	1167	938	2894.44
98	790	1167	1338	3294.44
99	790	1167	918	2874.55

100	790	1167	864	2820.26
101	790	1224	1224	3238.7
102	790	1224	1632	3646.5
103	790	1224	938	2952.14
104	790	1224	1338	3352.14
105	790	1224	918	2932.25
106	790	1224	864	2877.96
107	790	1632	1632	4054.3
108	790	1632	938	3359.94
109	790	1632	1338	3759.94
110	790	1632	918	3340.05
111	790	1632	864	3285.76
112	790	938	938	2665.58
113	790	938	1338	3065.58
114	790	938	918	2645.69
115	790	938	864	2591.4
116	790	1338	1338	3465.58

117	790	1338	918	3045.69
118	790	1338	864	2991.4
119	790	918	918	2625.8
120	790	918	864	2571.51
121	790	864	864	2517.22
122	1380	1380	1380	4139.7
123	1380	1380	1603	4362.45
124	1380	1380	1167	3926.45
125	1380	1380	1224	3984.15
126	1380	1380	1632	4391.95
127	1380	1380	938	3697.59
128	1380	1380	1338	4097.59
129	1380	1380	918	3677.7
130	1380	1380	864	3623.41
131	1380	1603	1603	4585.2
132	1380	1603	1167	4149.2
133	1380	1603	1224	4206.9

134	1380	1603	1632	4614.7
135	1380	1603	938	3920.34
136	1380	1603	1338	4320.34
137	1380	1603	918	3900.45
138	1380	1603	864	3846.16
139	1380	1167	1167	3713.2
140	1380	1167	1224	3770.9
141	1380	1167	1632	4178.7
142	1380	1167	938	3484.34
143	1380	1167	1338	3884.34
144	1380	1167	918	3464.45
145	1380	1167	864	3410.16
146	1380	1224	1224	3828.6
147	1380	1224	1632	4236.4
148	1380	1224	938	3542.04
149	1380	1224	1338	3942.04
150	1380	1224	918	3522.15

151	1380	1224	864	3467.86
152	1380	1632	1632	4644.2
153	1380	1632	938	3949.84
154	1380	1632	1338	4349.84
155	1380	1632	918	3929.95
156	1380	1632	864	3875.66
157	1380	938	938	3255.48
158	1380	938	1338	3655.48
159	1380	938	918	3235.59
160	1380	938	864	3181.3
161	1380	1338	1338	4055.48
162	1380	1338	918	3635.59
163	1380	1338	864	3581.3
164	1380	918	918	3215.7
165	1380	918	864	3161.41
166	1380	864	864	3107.12
167	1603	1603	1603	4807.95

168	1603	1603	1167	4371.95
169	1603	1603	1224	4429.65
170	1603	1603	1632	4837.45
171	1603	1603	938	4143.09
172	1603	1603	1338	4543.09
173	1603	1603	918	4123.2
174	1603	1603	864	4068.91
175	1603	1167	1167	3935.95
176	1603	1167	1224	3993.65
177	1603	1167	1632	4401.45
178	1603	1167	938	3707.09
179	1603	1167	1338	4107.09
180	1603	1167	918	3687.2
181	1603	1167	864	3632.91
182	1603	1224	1224	4051.35
183	1603	1224	1632	4459.15
184	1603	1224	938	3764.79

185	1603	1224	1338	4164.79
186	1603	1224	918	3744.9
187	1603	1224	864	3690.61
188	1603	1632	1632	4866.95
189	1603	1632	938	4172.59
190	1603	1632	1338	4572.59
191	1603	1632	918	4152.7
192	1603	1632	864	4098.41
193	1603	938	938	3478.23
194	1603	938	1338	3878.23
195	1603	938	918	3458.34
196	1603	938	864	3404.05
197	1603	1338	1338	4278.23
198	1603	1338	918	3858.34
199	1603	1338	864	3804.05
200	1603	918	918	3438.45
201	1603	918	864	3384.16

202	1603	864	864	3329.87
203	1167	1167	1167	3499.95
204	1167	1167	1224	3557.65
205	1167	1167	1632	3965.45
206	1167	1167	938	3271.09
207	1167	1167	1338	3671.09
208	1167	1167	918	3251.2
209	1167	1167	864	3196.91
210	1167	1224	1224	3615.35
211	1167	1224	1632	4023.15
212	1167	1224	938	3328.79
213	1167	1224	1338	3728.79
214	1167	1224	918	3308.9
215	1167	1224	864	3254.61
216	1167	1632	1632	4430.95
217	1167	1632	938	3736.59
218	1167	1632	1338	4136.59

219	1167	1632	918	3716.7
220	1167	1632	864	3662.41
221	1167	938	938	3042.23
222	1167	938	1338	3442.23
223	1167	938	918	3022.34
224	1167	938	864	2968.05
225	1167	1338	1338	3842.23
226	1167	1338	918	3422.34
227	1167	1338	864	3368.05
228	1167	918	918	3002.45
229	1167	918	864	2948.16
230	1167	864	864	2893.87
231	1224	1224	1224	3673.05
232	1224	1224	1632	4080.85
233	1224	1224	938	3386.49
234	1224	1224	1338	3786.49
235	1224	1224	918	3366.6

236	1224	1224	864	3312.31
237	1224	1632	1632	4488.65
238	1224	1632	938	3794.29
239	1224	1632	1338	4194.29
240	1224	1632	918	3774.4
241	1224	1632	864	3720.11
242	1224	938	938	3099.93
243	1224	938	1338	3499.93
244	1224	938	918	3080.04
245	1224	938	864	3025.75
246	1224	1338	1338	3899.93
247	1224	1338	918	3480.04
248	1224	1338	864	3425.75
249	1224	918	918	3060.15
250	1224	918	864	3005.86
251	1224	864	864	2951.57
252	1632	1632	1632	4896.45

253	1632	1632	938	4202.09
254	1632	1632	1338	4602.09
255	1632	1632	918	4182.2
256	1632	1632	864	4127.91
257	1632	938	938	3507.73
258	1632	938	1338	3907.73
259	1632	938	918	3487.84
260	1632	938	864	3433.55
261	1632	1338	1338	4307.73
262	1632	1338	918	3887.84
263	1632	1338	864	3833.55
264	1632	918	918	3467.95
265	1632	918	864	3413.66
266	1632	864	864	3359.37
267	938	938	938	2813.37
268	938	938	1338	3213.37
269	938	938	918	2793.48

270	938	938	864	2739.19
271	938	1338	1338	3613.37
272	938	1338	918	3193.48
273	938	1338	864	3139.19
274	938	918	918	2773.59
275	938	918	864	2719.3
276	938	864	864	2665.01
277	1338	1338	1338	4013.37
278	1338	1338	918	3593.48
279	1338	1338	864	3539.19
280	1338	918	918	3173.59
281	1338	918	864	3119.3
282	1338	864	864	3065.01
283	918	918	918	2753.7
284	918	918	864	2699.41
285	918	864	864	2645.12
286	864	864	864	2590.83

mean	3449.591
SD	558.0247
